# Never too late? An advantage on tests of auditory attention extends to late bilinguals

**DOI:** 10.3389/fpsyg.2014.00485

**Published:** 2014-05-26

**Authors:** Thomas H. Bak, Mariana Vega-Mendoza, Antonella Sorace

**Affiliations:** ^1^Department of Psychology, School of Philosophy, University of EdinburghEdinburgh, UK; ^2^Linguistics and English Language, School of Philosophy, University of EdinburghEdinburgh, UK

**Keywords:** bilingualism, cognition, attention, auditory attention, age of acquisition

## Abstract

Recent studies, using predominantly visual tasks, indicate that early bilinguals tend to outperform monolinguals on attention tests. It remains less clear whether such advantages extend to those bilinguals who have acquired their second language later in life. We examined this question in 38 monolingual and 60 bilingual university students. The bilingual group was further subdivided into early childhood (ECB), late childhood (LCB), and early adulthood bilinguals (EAB). The assessment consisted of five subtests from the clinically validated Test of Everyday Attention (TEA). Overall, bilinguals outperformed monolinguals on auditory attention tests, but not on visual search tasks. The latter observation suggests that the differences between bilinguals and monolinguals are specific and not due to a generally higher cognitive performance in bilinguals. Within the bilingual group, ECB showed a larger advantage on attention switching, LCB/EAB on selective attention. We conclude that the effects of bilingualism extend into the auditory domain and are not confined to childhood bilinguals, although their scope might be slightly different in early and late bilinguals.

## Introduction

For many decades, the study of bilingualism focused on the linguistic differences between monolingual and bilingual children and adults, such as vocabulary size, lexical access, and morphosyntactic development (see De Houwer, 2009, for a review). However, from the 1990s onward the idea emerged that the experience of bilingualism might also influence cognitive functions other than language. Studies comparing mono- and bilingual children suggested a bilingual advantage in non-verbal problem-solving tasks such as the dimensional change card sort task, cardinal quantity tasks, and, with particular relevance to the present study, in the control of attention (Frye et al., 1995; Zelazo et al., 1996; Zelazo and Frye, 1997; Bialystok, 1999; Bialystok and Martin, 2004).

Recent studies demonstrate that these differences persist well-beyond childhood (Bialystok et al., 2004, 2006, 2008, 2012). Using the Simon task (Simon and Small Jr, 1969), Bialystok et al. (2004) found that although the bilingual advantage was consistent between the ages of 30 and 60, after the age of 60 response times began to decrease in both monolinguals and bilinguals but this decline was significantly slower in the latter group. These cognitive advantages of bilingualism in older adults can be of considerable practical relevance, leading to a slower cognitive aging and a later onset of dementia (Bialystok et al., 2007). Indeed, studies from different countries, with radically different populations, cultures, and languages arrived at a remarkably similar estimate of a 4–5 years delay in the onset of dementia in bilingual patients when compared to monolinguals (Alladi et al., 2013). Thus, bilingualism is starting to play an increasingly important role in the current debates about cognitive reserve and the factors influencing cognitive aging and dementia (Bak and Alladi, 2014).

Different explanations have been put forward to account for these apparent cognitive differences between bilinguals and monolinguals. Kroll and De Groot (1997) postulate that the bilingual advantage results from a greater cognitive flexibility due to the need to select appropriate language options from one common conceptual store, which contains a large number of mappings of words and concepts. In contrast, Green (1998) argues that bilinguals have better inhibitory control because, in order to prevent ongoing interference, they must inhibit the language not in use. Indeed, a study by Treccani et al. (2009) demonstrates that the very efficiency of inhibitory processes in bilinguals can turn into a disadvantage when a new task requires activation of previously inhibited material. Other studies (Hilchey and Klein, 2011; Hernández et al., 2013) have recently questioned explanations of the bilingual advantage in terms of inhibitory control, calling for more in-depth research on different components of executive function and on the different operations of the central executive system. Some researchers have also questioned the generalizability of results showing a bilingual advantage, based on the heterogeneity of the bilingual population, the instability of these results and a number of failed attempts to replicate them (Paap and Greenberg, 2013; see Kroll and Bialystok, 2013 as response). While this debate is still open, the field is engaged in finding exactly how specific factors affecting the bilingual experience relate to specific components of executive control (Paap, 2014). The present study is a contribution to this wider aim.

One of the factors that might influence the nature of cognitive processing in bilinguals is the age of acquisition of the second language. Early studies of cognition in bilinguals focused on simultaneous or early successive bilinguals who acquired both languages in their first years of life and it is in this group that bilingual cognitive advantages have been best documented (Bialystok, 2007). However, recent studies suggest that both early and late bilingualism might have significant, yet different influence on frontal-executive functions, with early bilinguals being better at switching, late at inhibiting (Tao et al., 2011). Indeed, early and late bilingualism could be associated with different patterns of brain development (Klein et al., 2014). Given that the acquisition of a second language in adulthood is arguably becoming more common than the ideal case of early simultaneous bilingualism, it is important to determine whether the effects of bilingualism—advantageous or disadvantageous—extend to this population.

The identification of bilingualism as a potential factor delaying dementia (Bak and Alladi, 2014) brings a new set of challenges to the researchers working in this field. In order to explore the impact of bilingualism on healthy and on pathological aging, we need large studies, including healthy elderly population as well as patients suffering from different brain diseases. These types of participants require brief, easily applicable tests, ideally those already in use in clinical populations. In contrast, the majority of studies exploring cognitive differences between monolinguals and bilinguals so far have been using complex experimental paradigms applied in laboratory settings. Such procedures cannot be easily used in large cohorts of elderly participants, let alone in patients with dementia, stroke, head injury or other disorders affecting nervous system. What is needed, therefore, is a brief clinical instrument sensitive to potential cognitive differences between mono- and bilinguals.

The Test of Everyday Attention (TEA) (Robertson et al., 1994) offers a particularly suitable tool to address this problem. Firstly, it is a well-established and widely used clinical test, with large sets of normative data collected in healthy elderly Western (Robertson et al., 1996) and Asian (Chan et al., 2006) populations. Secondly, it has been successfully applied in a wide range of neurological diseases, including stroke, head injury, dementia, and other neurodegenerative conditions (Robertson et al., 1996; Chan, 2000; Chen et al., 2013). This means that the tasks are clear enough to be understood by those patient groups but, at the same time, sensitive enough to detect impairments. Thirdly, the TEA consists of different subtests, assessing different components of the attentional system: sustained attention, selective attention, and attentional switching (Robertson et al., 1996). It allows, therefore, a separate assessment of different forms of attention. Finally, while most cognitive test batteries tend to use predominantly visual material, which is generally easier to administer (Bak and Mioshi, 2007), the TEA has several auditory subtests based on tone counting (so called “Elevator tasks”).

The last aspect seemed to us to be of special interest in the context of bilingualism. In comparison with the wealth of studies examining the visual domain, much less is known about possible differences in auditory processing between mono- and bilinguals, despite the importance of the auditory domain in language acquisition and use. Moreover, the results of auditory studies of bilinguals and monolinguals have so far produced conflicting results. Bialystok and DePape (2009) did not find an advantage of bilinguals over monolinguals on an auditory Stroop task, while other authors reported a better performance in bilinguals on dichotic listening (Hamalainen and Hugdahl, 2011) and sound encoding (Krizman et al., 2012). Interestingly, the first study was based on non-linguistic stimuli (pitch), while the last two used as experimental material syllables such as “da” or “ba,” which form part of the sound repertoire of the languages in question. It is conceivable, therefore, that the linguistic nature of the stimuli provides an advantage for bilinguals. Hence, in order to establish whether the cognitive effects of bilingualism extend into the auditory domain, it is necessary to use tasks that minimize verbal elements as much as possible.

Based on these considerations, we have selected for our study five TEA subtests measuring different aspects of attention. Initially (Experiment 1), we selected the so-called Elevator Tasks 1–3, measuring in the auditory domain sustained attention (Elevator Task 1), selective attention (Elevator Task 2), and attentional switching (Elevator Task 3). Extending the results from the first experiment, we have added in Experiment 2 two further subtests (Telephone Search and Telephone Search while counting). These tasks assess visual search, an aspect of attention which, although demanding, does not require processing of conflicting information (e.g., switching, inhibition). Accordingly, we did not expect it to be influenced by bilingualism. These subtests can help, therefore, to determine whether possible differences between mono- and bilingual groups are due to general, differences in cognitive performance, or to specific aspects of attention. In Experiment 1, we examined early childhood bilinguals (ECB) (those who acquired both languages before the age of 4) and late childhood bilinguals (LCB) (who acquired the second language between the ages of 4 and 15 years). In Experiment 2, we extended the study to early adulthood bilinguals (EAB) (whose second language acquisition took place between the ages of 15 and 19).

## Methods

### Participants

#### Experiment 1

All 60 subjects were students at the University of Edinburgh, who understood and spoke English fluently. Based on the results of the Language Ability Questionnaire (see Appendix 1 in Supplementary Material), 19 were classified as monolinguals (ML), 23 as ECB, and 18 as LCB (see Appendix 2 for a detailed list of languages spoken by each participant). There were no significant differences in age or gender distribution between the groups—age: ML: 22.2 ± 1.6; ECB: 21.3 ± 1.7; LCB: 23.6 ± 4.3 and gender (percentage females): ML: 73%, ECB: 44%, LCB: 50%.

#### Experiment 2

All 38 subjects were also students at the University of Edinburgh with fluent command of English. None of them had participated in the Experiment 1. Based on the results of the same Language Ability Questionnaire as in Experiment 1, the group was split into 19 monolinguals (ML) and 19 EAB, who acquired their second language between the ages of 15 and 19 years. There were no significant differences in age or gender distribution between the groups—age: ML: 21.5 ± 1.0; EAB: 22.9 ± 3.3 and gender (percentage females): ML: 36%; EAB: 36%. The study has been approved by the Ethics Committee of the University of Edinburgh, Psychology Department.

### Assessment of attentional functions

Both experiments consisted of subtests for the TEA, a standardized test battery to assess attentional functions (Robertson et al., 1994). Experiment 1 consisted of three TEA subtests (Elevator Tasks 1–3). In Experiment 2 we have used identical procedure for the tests TEA 1–3, but continued with two further subtests (Telephone Search and Telephone Search Dual Task). The test was conducted in a quiet laboratory space, with instructions and tones presented from a tape using headphones.

#### Elevator counting (elevator task 1)

Subjects are asked to count simple tones of the same pitch and duration presented at irregular intervals; used as a measure of sustained attention.

#### Elevator counting with distraction (elevator task 2)

Subjects hear low and high tones and count the number of low tones while ignoring the high ones; used as a measure of selective attention.

#### Elevator counting with reversal (elevator task 3)

Subjects hear a sequence of three different tones: a middle-pitched, high, and low tone, they are asked to count the middle-pitched tones, upwards if preceded by a high, downwards if preceded by a low tone; used as a measure of attentional switching.

#### Telephone search

Subjects are given a telephone book directory page and a cue-book illustrating the target symbols. The task consists of circling all entries with a given combination of symbols.

#### Telephone search dual task

Same instructions as above, with the additional difficulty that the subjects had to conduct the task while at the same time counting auditorily presented tones (simple tones of the same pitch, as in the Elevator Task 1).

## Results

### Experiment 1

**Figure 1:**
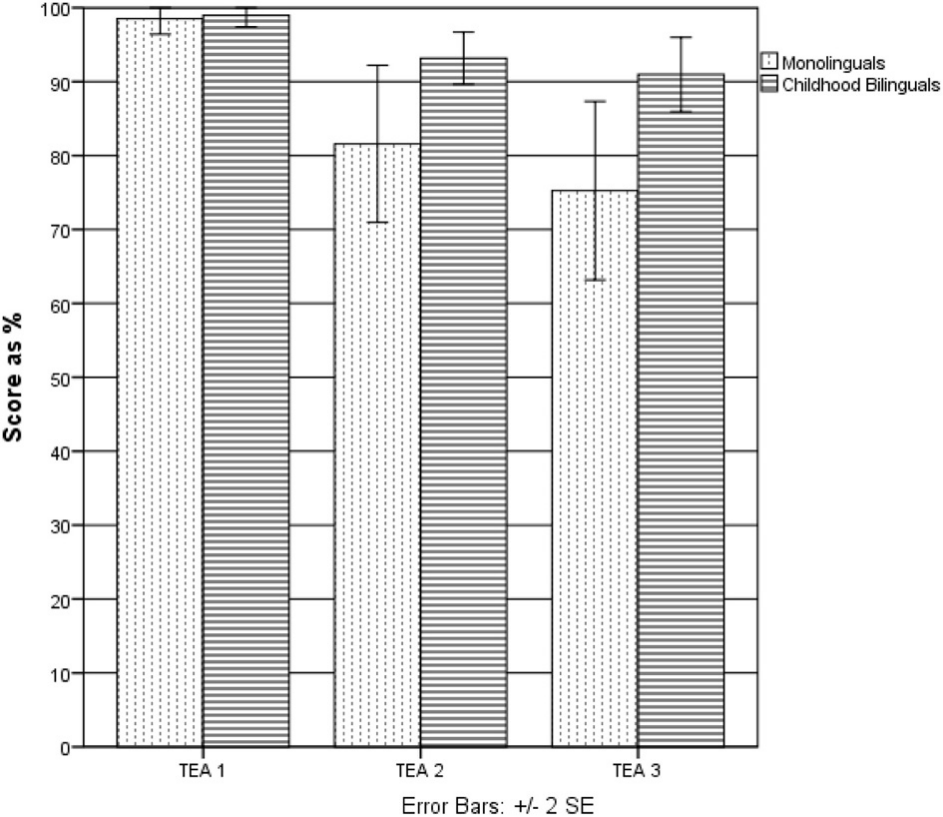
**Difference between the monolingual and childhood bilingual groups on TEA sub-tests (Experiment 1)**.

First, a comparison was conducted between the bilingual group as a whole (ECB and LCB) on one hand and the monolingual group on the other (see Figure 1 and Table 1). The Mann–Whitney test, used since the data were not normally distributed, revealed that bilinguals scored significantly higher than monolinguals in Elevator Task 2 (*U* = 466, *p* < 0.05) and Elevator Task 3 (*U* = 414.50, *p* < 0.05). No significant difference was observed on Elevator Task 1.

**Table 1:** **Experiment 1—Comparison of the number of correct answers in Monolinguals vs. Childhood bilinguals**.

	**Monolinguals (*N* = 19)**	**Childhood bilinguals (*N* = 41)**
Elevator task	6.89 ± 0.3	6.93 ± 0.2
Elevator task with distraction	8.16 ± 2.3	9.32 ± 1.0^*^
Elevator task with reversal	7.53 ± 2.6	9.09 ± 1.6^*^

* p < 0.05 vs. ML (Mann–Whitney-U-test).

**Figure 2:**
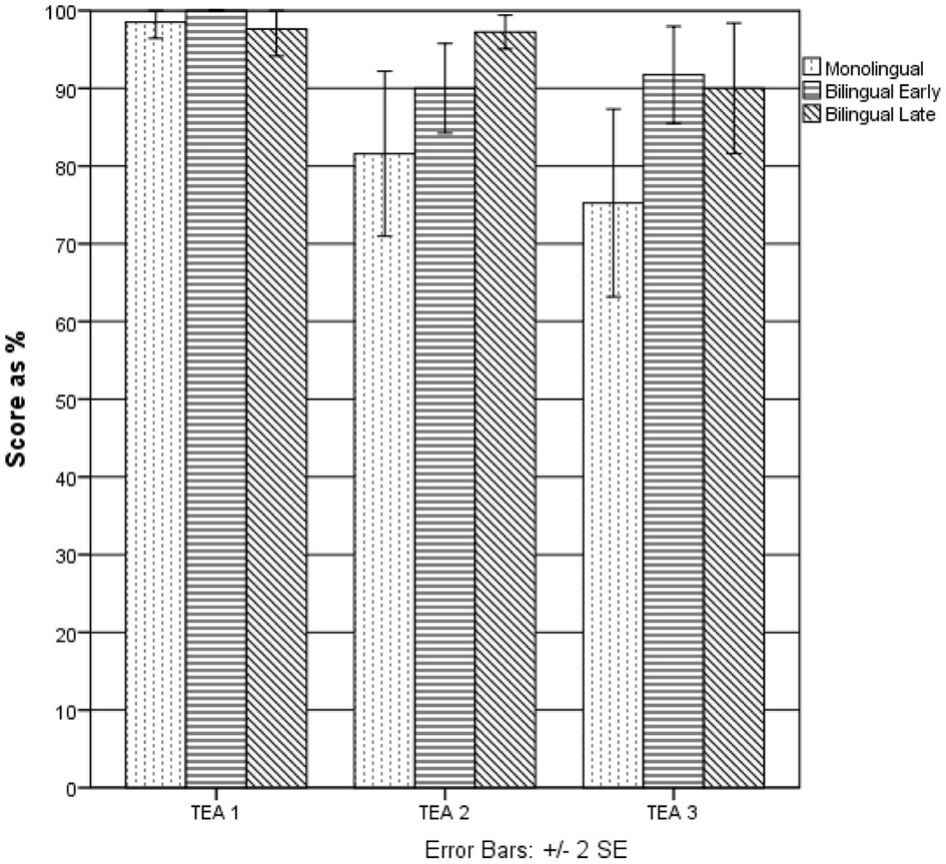
**Differences between the monolingual, the early, and the late childhood bilingual groups on TEA sub-tests (Experiment 1)**.

Subsequently, a Mann–Whitney test was performed to compare separately both bilingual groups with the monolingual one (see Table 2 and Figure 2). There were no significant differences between the groups in Elevator Task 1. In Elevator Task 2, LCB scored significantly higher than ML (*U* = 99.50, *p* < 0.05, *r* = −0.40), but no significant difference was observed between ECB and ML. In Elevator Task 3, in contrast, ECB scored significantly higher than ML (*U* = 103.50, *p* < 0.05, *r* = −0.35), while no significant difference was found between LCB and ML.

**Table 2:** **Experiment 1—Comparison of the number of correct answers in Monolinguals vs. Early and vs. Late childhood bilinguals**.

	**Monolinguals (*N* = 19)**	**Early childhood bilinguals (*N* = 23)**	**Late childhood bilinguals (*N* = 18)**
Elevator task	6.89 ± 0.3	7.00 ± 0.0	6.83 ± 0.5
Elevator task with distraction	8.16 ± 2.3	9.00 ± 1.4	9.72 ± 0.5^*^
Elevator task with reversal	7.53 ± 2.6	9.17 ± 1.5^*^	9.00 ± 1.8

* p < 0.05 vs. ML (Mann–Whitney-U-test).

### Experiment 2

**Figure 3:**
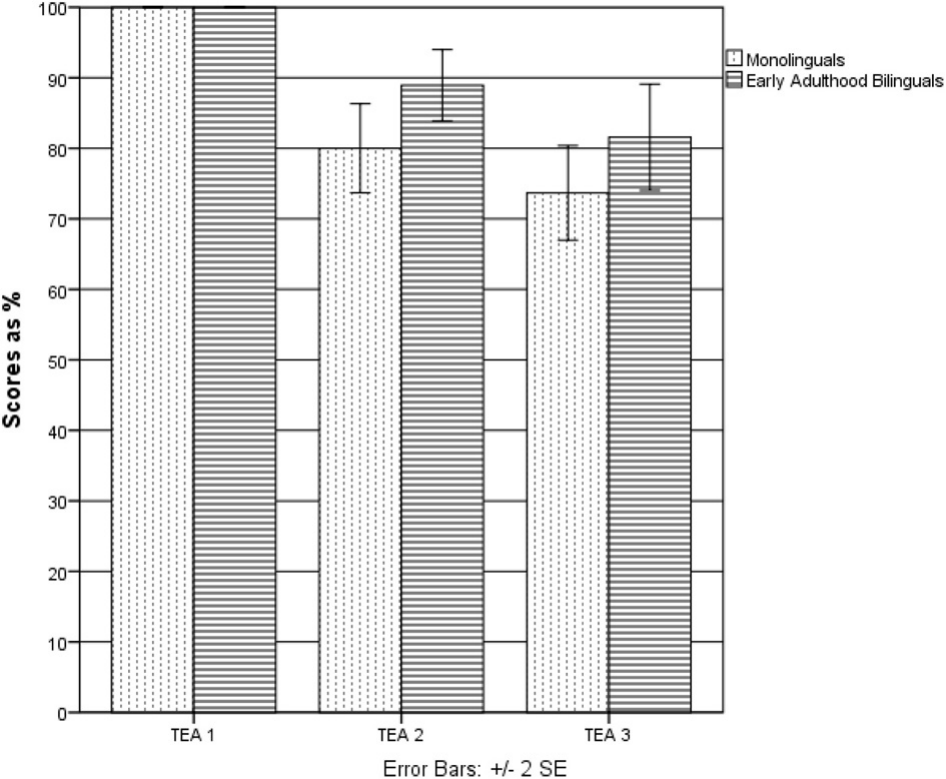
**Differences between the monolingual and early adulthood bilingual groups on TEA sub-tests (Experiment 2)**.

Since, as in Experiment 1, the data was not normally distributed, Mann–Whitney *U*-test was performed (see Table 3 and Figure 3). No significant differences were found on Elevator Task 1. On Elevator Task 2, EAB performed significantly better than ML (*U* = 109, *p* < 0.05). Although the EAB performed also slightly better than monolinguals also on Elevator Task 3, the difference did not reach significance level (*U* = 129, *p* = 0.13). No differences between the groups were observed in the Telephone Search (*U* = 154, *p* = 0.43) and Telephone Search Dual Task (*U* = 154.5, *p* = 0.44).

**Table 3:** **Experiment 2—Comparison of the number of correct answers in Monolinguals vs. Early adulthood bilinguals**.

	**Monolinguals (*N* = 19)**	**Early adult bilinguals (*N* = 19)**
Elevator task	7.00 ± 0.00	7.00 ± 0.00
Elevator task with distraction	7.94 ± 1.32	8.89 ± 1.07^*^
Elevator task with reversal	7.37 ± 1.42	8.16 ± 1.60
Telephone search	2.69 ± 0.96	3.02 ± 1.14
Telephone search dual task	3.28 ± 0.94	3.72 ± 1.38

* p < 0.05 vs. ML (Mann–Whitney-U-test).

## Discussion

In both experiments the performance on the subtests of the TEA revealed specific differences between the mono- and the bilingual group. The bilingual advantage on Elevator Tasks 2 and 3 confirms previous reports of bilingual advantage on cognitively demanding attentional control tasks (Bialystok et al., 2006; Treccani et al., 2009), extending them into the domain of auditory attention. The bilingual advantage was demonstrated using a relatively simple attentional task adapted from a standardized clinical assessment battery. In comparison with the sophisticated computerized design used in many previous studies, the TEA subtests have the advantage of easy applicability: they are fast, easy to perform and evaluate, do not require a lab setting and can be used in conjunction with any type of tape recorder or a laptop. Moreover, they are already used across the world in different clinical populations (Robertson et al., 1996; Chan, 2000; Chen et al., 2013). They could find, therefore, widespread use in future studies of cognitive functions in bilingualism, particularly in large cohort studies of cognitive aging and dementia (Bak and Alladi, 2014), in which current experimental paradigms would not be practicable.

Consistent with recent reports that qualify the scope of the monolingual-bilingual difference (Hilchey and Klein, 2011; Tao et al., 2011), the influence of bilingualism on attention observed in our study was selective, affecting specific cognitive functions. Bilingual groups were not uniformly better on all attentional tests included in this study. In Experiment 2, there was no difference between the groups on Telephone Search and Telephone Search Dual Task. Both are difficult and demanding tasks, in which none of the groups reached ceiling level. However, the type of attention required to perform a visual search required in Telephone Search and Telephone Search Dual Task is different in quality from selective attention of Elevator Task 2 and attention switching of Elevator Task 3. Even in Telephone Search Dual Task which includes a dual task (simultaneous visual search and counting tones), both tasks involve different modalities (visual and auditory) and are, therefore, fundamentally different from the experience of bilingualism, in which the selection and switching normally happen within the same modality (except in bimodal bilingualism; Emmorey et al., 2008a,b). Furthermore, the fact that the bilingual advantage is confined to Elevator Task 2 and 3 and does not seem to affect Telephone Search and Telephone Search Dual Task suggests that this effect is not easily explained by a sample bias, such as a higher general intelligence or an overall better level of cognitive performance in the bilingual group.

Importantly, the effects of the bilingual experience were not confined to ECB. It was observed in all three groups characterized by different age of acquisition of the second language (early and late childhood and early adulthood). Traditionally, the majority of bilingualism studies has focused on speakers who acquired both languages in the first years of life, during the period of maximal sensitivity to language stimuli (or “Critical Period”; see Birdsong, 1999; Newport et al., 2001). However, many people start learning a second language in late childhood or adulthood and reach a very high and even native-like level of proficiency (Sorace, 2004; Sorace and Filiaci, 2006; MacLeod and Stoel-Gammon, 2010). The question whether this large group can also benefit from cognitive effects of bilingualism is of considerable practical relevance, particularly in light of the recent findings about the dementia-delaying effects of bilingualism (Alladi et al., 2013; Bak and Alladi, 2014).

However, although we found a positive effect of bilingualism in all three groups we examined (early and late childhood, early adulthood), its mechanisms might be slightly different. In a recent study, comparing early and late onset bilinguals, only those who started using both languages before the age of 10 were found to have a cognitive advantage (Luk et al., 2011). In contrast, Tao et al. (2011) found that both, early and late bilingual groups benefitted from bilingualism, but in different ways: the early group mainly on switching, the late on inhibition. Our results would be in line with this hypothesis. The cognitive requirements of the Elevator Tasks 2 and 3 are slightly different: Elevator Task 2 requires selective attention and successful inhibition of irrelevant stimuli. It could be compared, therefore, to visual inhibition tasks such as those used by Treccani et al. (2009). Elevator Task 3, in contrast, involves attentional switching between two (unpredictable) directions of counting. Hence, it is more similar to visual paradigms used by Prior and MacWhinney (2010) and Costa et al. (2009). It seems plausible that the early childhood experience of two languages especially enhances switching processes, whereas the later acquisition of a second language after the consolidation of the first one might require stronger inhibition of the native dominant language and would therefore have a greater impact on inhibitory control.

Our study has limitations. We have not conducted a general assessment of cognitive abilities beyond the TEA subtests used in our protocol. Although the dissociation between the Elevator Tasks, with a bilingual advantage and the Telephone Search Tasks without it speaks in favor of a specific effect of bilingualism on cognition we cannot exclude the possibility of “reverse causality.” It could be that it is not bilingualism that causes a cognitive advantage in certain individuals but that a superior baseline cognitive ability makes them more likely to acquire more than one language. Such confound is extremely difficult to address, since it would require either longitudinal studies or at least knowledge of baseline cognitive abilities in early childhood. Since this is the first time that the TEA has been used not to characterize brain diseases but individual variations in performance in normal population depending on the knowledge of languages, we do not know which practical consequences the observed differences might produce. Finally, we have not examined individuals who acquired a second language after the age of 19 years—a large and very important group.

However, we hope that by raising the question whether a bilingualism advantage can be observed well-beyond the traditional age boundaries of critical periods, our study will encourage further research, using a wider range of tasks and comparing groups of subjects who acquired the second language at different stages of their lives. In this way, we might be able to determine exactly which aspects of cognitive processing are affected by the bilingual experience and whether age of onset of bilingualism might have differential impacts on cognitive functions. The fact that these questions can be addressed using a brief and simple standardized cognitive tests such as TEA makes this field of study all the more promising.

### Conflict of interest statement

The authors declare that the research was conducted in the absence of any commercial or financial relationships that could be construed as a potential conflict of interest.
