# Supplementary material for: Never too late? An advantage on tests of auditory attention extends to late bilinguals
Source: Front Psychol. 2014 May 26;5:485. doi: 10.3389/fpsyg.2014.00485 (PMC4033267; doi:10.3389/fpsyg.2014.00485)
Supplement: Supplementary file 1 [file DataSheet1.DOCX]

**Appendix 1: Language Ability Questionnaire**

**Demographics:**

1. Gender: **M / F**
2. Age ___
3. If student, which department? _____________________
4. If not student, what is your occupation? ______________________
5. Primary email address: __________________________

**Languages:**

In the box below, please list your languages in order of competence, starting with your strongest language, the age at which you started learning that language, and where/how it was acquired (an example is shown):


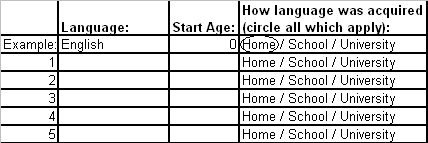


**Language Proficiency:**

On a scale of 1-5, please rate your language skills in the following areas for each language listed above: **1** – Basic; **2** – Weak; **3** – Moderate; **4** – Advanced; **5** – Fluent

|  | **Language:** | **Comprehension** | **Speaking** | **Reading** | **Writing** |
| --- | --- | --- | --- | --- | --- |
| Example: | English | **5** | **5** | **4** | **3** |
| 1 |  |  |  |  |  |
| 2 |  |  |  |  |  |
| 3 |  |  |  |  |  |
| 4 |  |  |  |  |  |
| 5 |  |  |  |  |  |

**Language Use Frequency:**

Do you ever use a language other than your first language? **Yes / No**

If you do, please rate your language use frequency in the following areas (exclude your first language): **a**) daily; **b**) weekly; **c**) monthly; **d**) yearly; **e**) never

|  | **Language:** | **Comprehension / Listening** | **Speaking** | **Reading** | **Writing** |
| --- | --- | --- | --- | --- | --- |
| Example: | French | **b** | **d** | **c** | **e** |
| 1 |  |  |  |  |  |
| 2 |  |  |  |  |  |
| 3 |  |  |  |  |  |
| 4 |  |  |  |  |  |
| 5 |  |  |  |  |  |

Do you have any auditory or visual impairment? **Yes / No**

If so, please specify____________________________

As far as you are aware, have you ever suffered any brain damage? **Yes / No**

If so, please describe briefly _____________________

Would you consider yourself (please circle):

a) monolingual

b) monolingual with basic knowledge of a foreign language(s) (e.g. briefly learned at school)

c) bilingual from birth (0-3 years) (i.e. acquired at home)

d) later bilingual/multilingual (acquired through formal teaching)

e) other (please specify) _________________________

**Appendix 2**

List of languages in which bilingual subjects reported proficiency.

A. Participants from Experiment 1

| **L1** | **L2** | **L3** | **L4** | **L5** |
| --- | --- | --- | --- | --- |
| English | Gujarati | French |  |  |
| Spanish | French | English |  |  |
| English | German |  |  |  |
| English | French |  |  |  |
| English | French |  |  |  |
| English | French |  |  |  |
| English | Bengali |  |  |  |
| Portuguese | English | Spanish | Italian | French |
| Russian | Polish | English | Spanish |  |
| English | Arabic |  |  |  |
| Sinhalese | Tamil | English | Italian |  |
| English | Cantonese |  |  |  |
| English | French |  |  |  |
| Greek | Finnish | German | English | Swedish |
| English | French |  |  |  |
| English | Arabic |  |  |  |
| English | Portuguese | Spanish |  |  |
| English | French |  |  |  |
| French | English |  |  |  |
| English | Swedish |  |  |  |
| English | Arabic |  |  |  |
| Hungarian | English | Spanish |  |  |
| English | French | Slovene |  |  |
| English | Welsh | German |  |  |
| Polish | English | French |  |  |
| Dutch | English | Spanish |  |  |
| German | English |  |  |  |
| Italian | English |  |  |  |
| English | Finnish |  |  |  |
| French | English |  |  |  |
| English | Italian | Finnish |  |  |
| Mandarin | Taiwanese | English |  |  |
| French | English |  |  |  |
| English | German |  |  |  |
| Spanish | Catalan | English |  |  |
| English | French |  |  |  |
| English | Irish | French |  |  |
| German | English |  |  |  |
| Polish | English |  |  |  |
| English | Cantonese |  |  |  |
| Portuguese | French | English |  |  |

B. Participants from Experiment 2.

| **Lang. 1** | **Lang. 2** | **Lang. 3** |
| --- | --- | --- |
| Persian | English |  |
| Chinese | French | English |
| Russian | English | Arabic |
| Tamil | English |  |
| Czech | German | English |
| English | Russian |  |
| English | German | Norwegian |
| Norwegian | English | Russian |
| English | Spanish | Russian |
| English | German |  |
| English | German |  |
| English | German |  |
| Spanish | English |  |
| English | Norwegian |  |
| English | German | Swedish |
| English | Norwegian |  |
| English | Swedish |  |
| English | Spanish |  |
| Polish | English |  |
